# Coupling of Action-Perception Brain Networks during Musical Pulse Processing: Evidence from Region-of-Interest-Based Independent Component Analysis

**DOI:** 10.3389/fnhum.2017.00230

**Published:** 2017-05-09

**Authors:** Iballa Burunat, Valeri Tsatsishvili, Elvira Brattico, Petri Toiviainen

**Affiliations:** ^1^Department of Music, Arts and Culture Studies, Finnish Centre for Interdisciplinary Music Research, University of JyväskyläJyväskylä, Finland; ^2^Department of Mathematical Information Technology, University of JyväskyläJyväskylä, Finland; ^3^Department of Clinical Medicine, Center for Music in the Brain, Aarhus University and The Royal Academy of Music Aarhus/AalborgAarhus, Denmark

**Keywords:** functional magnetic resonance imaging (fMRI), Independent Component Analysis (ICA), rhythm perception, musicians, naturalistic, prediction

## Abstract

Our sense of rhythm relies on orchestrated activity of several cerebral and cerebellar structures. Although functional connectivity studies have advanced our understanding of rhythm perception, this phenomenon has not been sufficiently studied as a function of musical training and beyond the General Linear Model (GLM) approach. Here, we studied pulse clarity processing during naturalistic music listening using a data-driven approach (independent component analysis; ICA). Participants' (18 musicians and 18 controls) functional magnetic resonance imaging (fMRI) responses were acquired while listening to music. A targeted region of interest (ROI) related to pulse clarity processing was defined, comprising auditory, somatomotor, basal ganglia, and cerebellar areas. The ICA decomposition was performed under different model orders, i.e., under a varying number of assumed independent sources, to avoid relying on prior model order assumptions. The components best predicted by a measure of the pulse clarity of the music, extracted computationally from the musical stimulus, were identified. Their corresponding spatial maps uncovered a network of auditory (perception) and motor (action) areas in an excitatory-inhibitory relationship at lower model orders, while mainly constrained to the auditory areas at higher model orders. Results revealed (a) a strengthened functional integration of action-perception networks associated with pulse clarity perception hidden from GLM analyses, and (b) group differences between musicians and non-musicians in pulse clarity processing, suggesting lifelong musical training as an important factor that may influence beat processing.

## Introduction

Pulse may be defined as an endogenous periodicity, a series of regularly recurring, precisely equivalent psychological events that arise in response to a musical rhythm (Cooper and Meyer, 1960; Large and Snyder, 2009). Although rhythms in music do not hold one-to-one relationships with auditory features (Kung et al., 2013), humans are able to effortlessly perceive the pulse in music. This phenomenon keeps challenging cognitive scientists, who pursue understanding of its underlying brain processes (Gabrielsson, 1987; Clarke, 1989; Palmer, 1989; Repp, 1990). This unique ability to perceive pulse allows us to coordinate motor movements to an external auditory stimulus (such as in music-induced foot tapping or dancing). Moving in synchrony with the beat (i.e., the predictive, perceived pulse in music; Patel, 2014) is in fact one of the most intriguing effects of music and a spontaneous behavior which has long puzzled scientists (Zentner and Eerola, 2010; Repp and Su, 2013). Furthermore, rhythm perception is fundamental to the experience of music and thus key for explaining musical behavior (Large and Palmer, 2002; Large and Snyder, 2009).

Beat perception in auditory rhythms is underpinned by interactions between activity in the auditory and motor systems (Zatorre et al., 2007; Grahn, 2009; Kung et al., 2013), which in particular may drive the temporal predictions involved in rhythm perception (Zatorre et al., 2007; Patel and Iversen, 2014). Recent fMRI evidence indicates that listening to salient rhythms in the absence of any overt movement recruits a cortico-subcortical functional network consisting of auditory cortex, premotor cortex (PMC), putamen (PUT), and supplementary motor area (SMA; Grahn and Rowe, 2009). In addition to the SMA and PMC, the cerebellum (CER) has been found to be active while listening to rhythms (Chen J. L. et al., 2008). Moreover, musical training seems to enhance auditory-motor coupling at the cortical level during rhythm processing (Chen et al., 2006; Grahn and Rowe, 2009), which is in line with evidence indicating that musicians show better rhythm synchronization than controls (Chen J. et al., 2008), likely due to a stronger internal representation of the beat or enhanced working memory abilities (Zatorre et al., 2010; Kung et al., 2011).

Connectivity studies have thus provided insights by exploring internal brain dependencies related to rhythm perception as modulated by musical training. They have, however, exclusively examined this phenomenon within the General Linear Model (GLM) approach, which allows studying brain activity as modeled by the researcher. In contrast, data-driven analyses require no explicit model of the temporal course of the brain activations, allowing for a more open, and comprehensive understanding of the brain mechanisms underlying rhythm processing. A well-studied data-driven approach is Independent Component Analysis (ICA), a blind source separation technique for studying networks on which we have no prior information. ICA is intrinsically a multivariate approach, i.e., it considers the relationships between all voxels simultaneously. Thus, it can provide an alternative and complementary approach to voxel-wise analyses. ICA can separate fMRI data into independent components (ICs), each of which represents spatially independent but functionally connected brain networks. What is special and interesting about ICA is that (a) it allows us to study connectivity and find networks without the need to rely on seed-based analysis, (b) it is a completely data-driven approach able to identify brain activity without a-priori assumptions of its dynamics; and (c) it has been applied reliably in naturalistic approaches stimuli (Bartels and Zeki, 2004, 2005; Malinen et al., 2007; Wolf et al., 2010), so complex naturalistic data can be analyzed reliably with consistent results.

In the current study, we aimed to identify the brain networks that respond to clarity of the pulse during music listening. The clarity or salience of the pulse is considered a high-level musical dimension that conveys how easily listeners can perceive the underlying metrical pulsation in a given musical piece (Lartillot et al., 2008). To study this phenomenon, we used a region-of-interest-based ICA (ROI-based ICA) approach. ROI-based ICA improves the separation and anatomical precision of the identified components that represent sources of interest, since (a) the brain volume does not affect the number of obtained components, and (b) informative signals with respect to potentially interesting sources are included in the analysis, thus excluding contributions otherwise used to separate non-interesting processes (e.g., artifacts; Formisano et al., 2004; Sohn et al., 2012; Beissner et al., 2014). To this end, we presented listeners (professional musicians and controls) with three pieces of music in randomized order while their fMRI responses were recorded. A targeted, hypothesis-driven subset of regions related to rhythm processing was included in the analysis, comprising cerebral and cerebellar areas: cortical auditory, motor and somatosensory regions of the cerebrum, cerebellar regions and, subcortically, the basal ganglia. Following a two-stage dimensionality reduction approach, ICA was applied in order to decompose participants' brain responses into spatially ICs. The ICA decomposition was performed under a range of model orders (e.g., dimensionality levels), namely, assuming different numbers of sources. The justification for this approach is that different choices of model order lead to the identification of different networks or subdivisions of networks (Abou-Elseoud et al., 2010; Kalcher et al., 2012). Component selection was based on the highest correlation coefficient between the associated temporal course and a continuous measure of the pulse clarity of the music. Additionally, GLM analyses of the data were performed as a complementary approach for comparison purposes. We expected to observe group differences in pulse clarity processing as a result of musicians' improved models of beat induction, evidenced in previous work using tapping paradigms (Drake et al., 2000; Aschersleben, 2002; Hove et al., 2007; Repp and Doggett, 2007; Krause et al., 2010; Repp, 2010). Because signal sources tend to merge into individual ICs in low models whereas they split into several subcomponents at high model orders (Abou-Elseoud et al., 2010), we additionally hypothesized that large-scale networks underpinning pulse clarity would be observed at low model orders, reflecting a scattered functional network previously reported in studies investigating rhythm processing. Accordingly, subcomponents of the large-scale networks that respond to pulse clarity would be observed at high model orders.

## Materials and methods

### Participants for the fMRI experiment

Thirty-six healthy participants with no history of neurological or psychological disorders participated in the fMRI experiment. The participants were screened for inclusion criteria before admission to the experiment (no ferromagnetic material in their body; no tattoo or recent permanent coloring; no pregnancy or breastfeeding; no chronic pharmacological medication; no claustrophobia). The participant pool was selected to include an equal number of professional musicians (*n* = 18, age = 28.2 ± 7.8, females = 9) and non-musicians (*n* = 18, age = 29.2 ± 10.7, females = 10, left-handers = 1). The criteria for musicianship was having more than 5 years of music training, having finished a music degree in a music academy, reporting themselves as musicians, and working professionally as a performer. As for the type of musicians, there were classical (*n* = 12), jazz (*n* = 4), and pop (*n* = 2) musicians. The instruments played were strings (violin = 4; cello = 2; double bass = 1), piano (*n* = 8), winds (trombone = 1; bassoon = 1), and mixed (*n* = 1). The musicians' group was homogeneous in terms of the duration of their musical training, onset age of instrument practice, and amount of years of active instrument playing. These details were obtained and crosschecked via questionnaires and HIMAB (Gold et al., 2013; Helsinki Inventory for Music and Affect Behavior). Both groups were comparable with respect to gender, age distribution, cognitive performance, socioeconomic status, and personality and mood questionnaire. The experiment was undertaken with the understanding and written consent of all participants. The study protocol proceeded upon acceptance by the ethics committee of the Coordinating Board of the Helsinki and Uusimaa Hospital District. The present dataset was part of a broad project (“Tunteet”) investigating different hypotheses related to auditory processing and its dependence on person-related factors by means of a multidimensional set of paradigms and tests, involving several experimental sessions, brain and behavioral measures as well as questionnaires. The findings related to the various hypotheses investigated appear in separate papers (cf. Alluri et al., 2015, 2017; Burunat et al., 2015, 2016; Kliuchko et al., 2015).

### Stimuli

The musical pieces used in the experiment were the following: (a) Stream of Consciousness by Dream Theater; (b) Adios Nonino by Astor Piazzolla; and (c) Rite of Spring (comprising the first three episodes from Part I: Introduction, Augurs of Spring, and Ritual of Abduction) by Igor Stravinsky. These are a progressive rock/metal piece, an Argentinian New Tango, and an iconic twentieth century classical work, respectively, thus covering distinct musical genres and styles. All three selected pieces are instrumental and have a duration of about 8 min. The recording details, musical excerpts used, and Spotify links to the musical stimuli can be found as Supplementary Material.

### fMRI experimental procedure

Participants' brain responses were acquired while they listened to each of the musical stimuli in a counterbalanced order. For each participant the stimuli loudness was adjusted to a comfortable but audible level inside the scanner room (around 75 dB). In the scanner, participants' only task was to attentively listen to the music delivered via high-quality MR-compatible insert earphones while keeping their eyes open.

### fMRI scanning and preprocessing

Scanning was performed using a 3T MAGNETOM Skyra whole-body scanner (Siemens Healthcare, Erlangen, Germany) and a standard 20-channel head-neck coil, at the Advanced Magnetic Imaging (AMI) Centre (Aalto University, Espoo, Finland). Using a single-shot gradient echo planar imaging (EPI) sequence, 33 oblique slices (field of view = 192 × 192 mm; 64 × 64 matrix; slice thickness = 4 mm, interslice skip = 0 mm; echo time = 32 ms; flip angle = 75°) were acquired every 2 s, providing whole-brain coverage. T1-weighted structural images (176 slices; field of view = 256 × 256 mm; matrix = 256 × 256; slice thickness = 1 mm; interslice skip = 0 mm; pulse sequence = MPRAGE) were also collected for individual coregistration. Functional MRI scans were preprocessed on a Matlab platform using SPM8 (Statistical Parametric Mapping), VBM5 for SPM (Voxel Based Morphometry; Ashburner and Friston, 2000); Wellcome Department of Imaging Neuroscience, London, UK), and customized scripts developed by the present authors. For each participant, low-resolution images were realigned on six dimensions using rigid body transformations (translation and rotation corrections did not exceed 2 mm and 2°, respectively), segmented into gray matter, white matter, and cerebrospinal fluid, and registered to the corresponding segmented high-resolution T1-weighted structural images. These were in turn normalized to the MNI (Montreal Neurological Institute; Evans et al., 1994) segmented standard a priori tissue templates using a 12-parameter affine transformation. Functional images were then blurred to best accommodate anatomical and functional variations across participants as well as to enhance the signal-to-noise by means of spatial smoothing using an 8 mm full-width-at-half-maximum Gaussian filter. Movement-related variance components in fMRI time series resulting from residual motion artifacts, assessed by the six parameters of the rigid body transformation in the realignment stage, were regressed out from each voxel time series. Next, spline interpolation was used to detrend the fMRI data, followed by temporal filtering (Gaussian smoothing with kernel width = 4 s).

Brain responses to the three stimuli were concatenated making a total of ~24 min worth of data. The rationale behind this was to combine stimuli representing a wide range of musical genres and styles in order to cancel out effects that the specific kinds of music may have on the phenomenon under investigation. The final time series had 702 samples after the four first samples of each of the three runs were removed to avoid artifacts due to magnetization effects.

### Region of interest (ROI) selection

**Figure 1:**
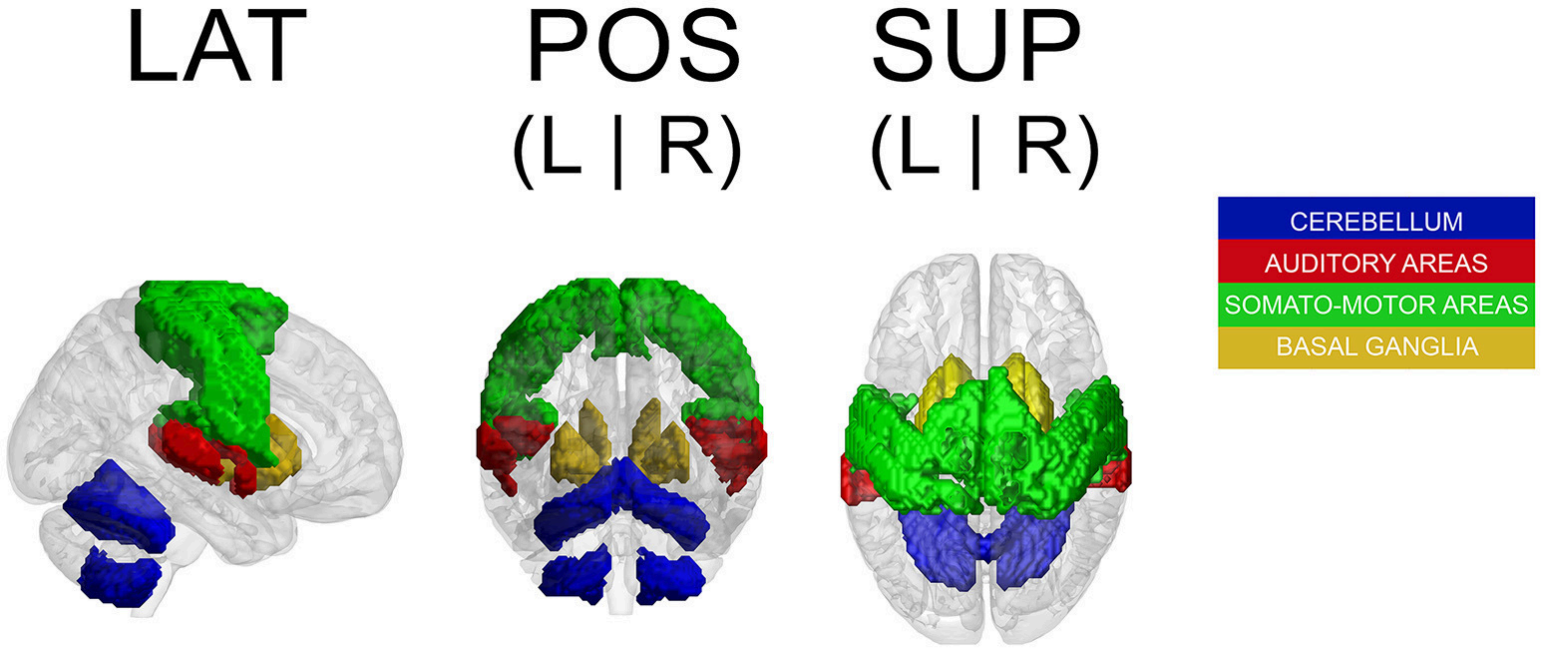
**Map of selected ROI related to rhythm processing**. LAT, lateral view; POS, posterior view; SUP, superior view; L, left; R, right.

Because a ROI-based ICA approach improves the separation and anatomical precision of the identified spatial components (Formisano et al., 2004; Sohn et al., 2012; Beissner et al., 2014), we only included in the analysis regions that have been identified in previous research as relevant in pulse processing. Previous studies show substantial overlap of neural substrates underlying rhythm processing, namely auditory cortices, PMC, SMA, CER, and the BG (Schubotz and von Cramon, 2001; Mayville et al., 2002; Ullén et al., 2002; Lewis et al., 2004; Grahn and Brett, 2007; Chen J. L. et al., 2008; Bengtsson et al., 2009; Grahn, 2012). Those ROIs were consequently included in the analysis with the addition of other potentially interesting areas, such as the primary motor cortex (M1), primary and secondary somatosensory cortices (S1 and S2, respectively), and rolandic operculum (ROper). The cerebellar regions included were lobules V, VI, and VIII (lobV, lobVI, lobVIII, respectively), previously associated with motor control (Penhune et al., 1998; Salmi et al., 2010; Bernard and Seidler, 2013) and rhythm processing (Grahn and Brett, 2007; Chen J. L. et al., 2008; Bengtsson et al., 2009; Grahn and McAuley, 2009). The ROI contained a total of 25,047 voxels (see Figure 1 for a map of selected ROI).

### Dual PCA reduction

Multi-subject ICA approaches are generally used in combination with dimensionality reduction methods in order to reduce the complexity for the subsequent ICA decomposition and avoid overfitting (Calhoun et al., 2001; Beckmann and Smith, 2005). Typically, dimension reduction is applied at both the individual and group levels. Performing subject-level principal component analysis (PCA) has the computational advantage of both reducing the dimensions of the data and denoising due to projecting the data onto their principal subspace. A second PCA at the group level is necessary prior to ICA to reduce the dimension of the data to the number of desired components estimated via ICA (Erhardt et al., 2011). This is required because the high dimensionality of the data from all subjects violates the ICA assumption of the determined mixture, where the number of fMRI images (mixtures) and sources match. Let *Y*_*i*_ denote the preprocessed, spatially normalized *T*-by- *V* data matrix for subject *i* out of *M* subjects, where *T* = time points (fMRI scans collected during the course of the experiment) and *V* = voxels. *Y*_*i*_ is subjected to PCA, resulting in the *L*-by-*V* PCA reduced data,

(1) $$\begin{array}{l} Y_{i}^{*}=\;F_{i}^{-}Y_{i}, \end{array}$$

where $F_{i}^{-}$ is the *L*-by-*T* reducing matrix of *L* number of principal components retained per subject. *L* is preferably chosen as a common value for all $F_{i}^{-}$, *i* = 1, …, *M* rather than separately for each $F_{i}^{-}$. The reason for this is that once in the back-reconstruction stage of subject-specific ICs, each subject has the same number of components determined by the ICA parameters.

**Figure 2:**
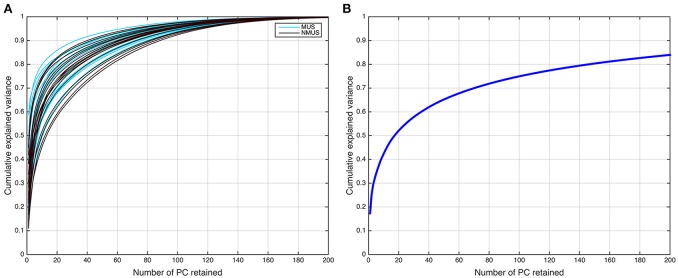
**Dual PCA reduction**. **(A)** Cumulative variance explained as a function of principal components (PCs) retained for the subject-level and **(B)** group-level PCA.

**Figure 3:**
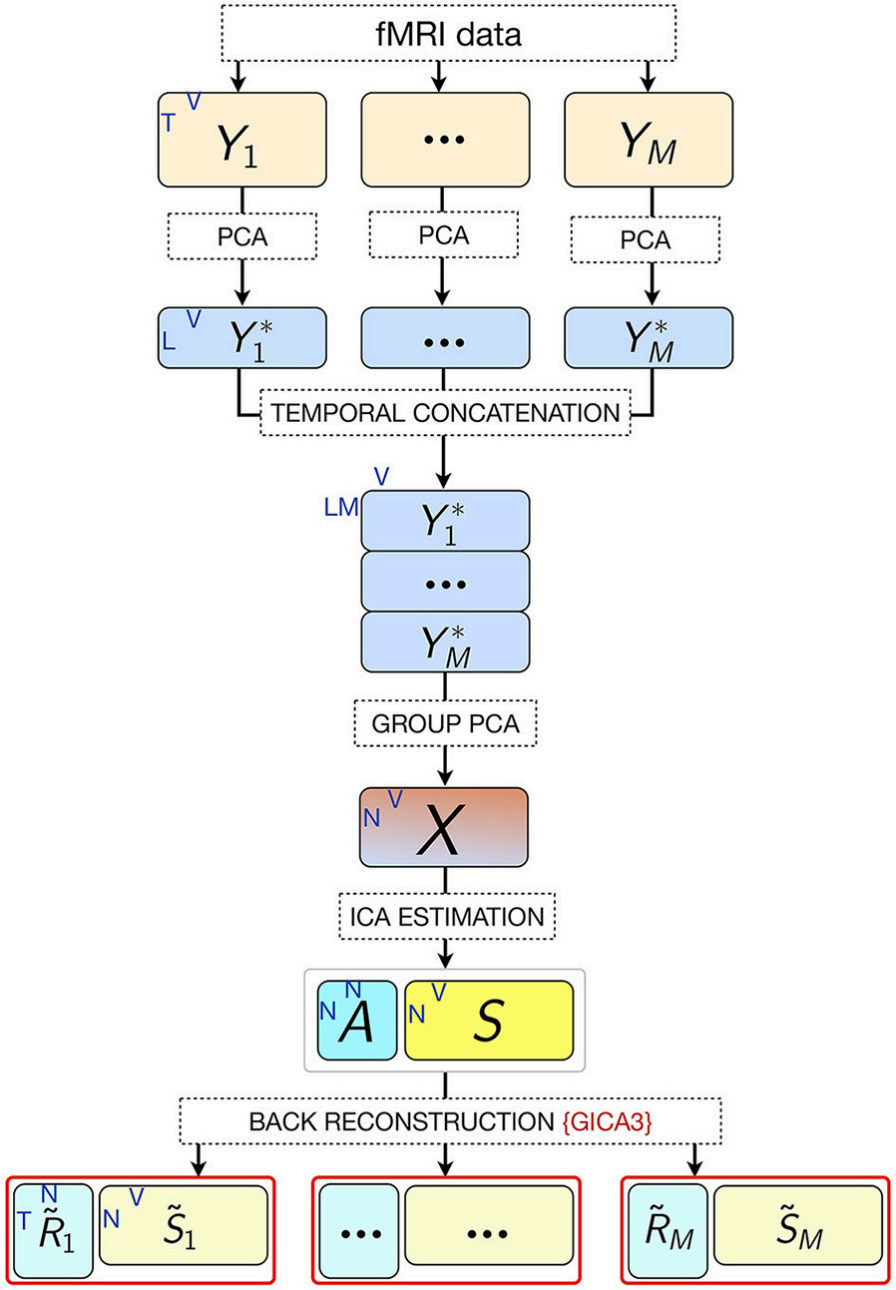
**Pipeline of the ICA approach**. M, number of participants; L, size of subject-level PCA reduced time dimension; K, number of fMRI time points; V, number of voxels; N, number of estimated ICs (model order).

Accordingly, the first 80 eigenvectors were retained in the subject-level PCA (*L* = 80), which preserved ~93% of the variance for each participant. The subject-level dimensions were thus reduced from 702 to 80 time points per participant. Following this, the PCA-reduced subject data were concatenated in the temporal domain for all 36 participants into an *LM*-by-*V* aggregate data matrix $\;Y^{*}={\left[Y_{1}^{*T},.\;.\;.,\;Y_{M}^{*T}\right]}^{T}$, which for our dataset was 2,880-by-25,047 (*LM* = 80 × 36 = 2, 880 concatenated time dimensions). The aggregate data were further reduced in a second PCA (group PCA) prior to ICA to *N*, the number of components to be estimated. Thus, the *N*-by- *V* group PCA-reduced matrix *X* was obtained,

(2) $$\begin{array}{l} X\equiv \;G^{-}Y^{*}=\left[G_{1}^{T},.\;.\;.\;,\;G_{M}^{T}\right]\left[\begin{array}{c} F_{1}^{-}Y_{1}\\ \vdots \\ F_{M}^{-}Y_{M} \end{array}\right] \end{array}$$

where *G* is the *LM*-by-*N* group PCA reducing matrix, and *G*^−^ denotes its pseudo-inverse (see Figure 2 for the variance as a function of number of principal components retained for both subject and group-level PCAs; and Figure 3 for overall ICA pipeline).

### The question of optimal model order selection

The selection of the optimal *N* or ICA model order to analyze fMRI data is not an easy problem and is still a subject of ongoing debate (Manoliu et al., 2013). This is because of the lack of a priori knowledge about the ground truth of the underlying components for brain imaging data and their modulation profiles across subjects. Several rules of thumb on an upper bound for model order estimation have been suggested for robust estimation of number of sources (ICs; Särelä and Vigário, 2003; Onton and Makeig, 2006), which suggest that model orders above a certain upper bound are expected to deteriorate ICA decomposition quality. According to the first of these rules (Särelä and Vigário, 2003), for robust estimation of *N* parameters (ICs) one needs *V* = 5 × *N*^2^, where *V* = samples (voxels). According to this rule of thumb, the upper limit in our dataset (*V* = 25, 047) corresponds to 70 ICs, explaining ~70% of the variance. However, the second rule (Onton and Makeig, 2006) suggests that the number of data points needed to find *N* stable ICs from ICA is typically *V* = *kN*^2^, where *k* is a multiplier with a recommended value of *k* ≥ 25. Accordingly, the decomposition quality in our dataset would start to deteriorate above a model order of ~35 ICs. It should be noted, however, that these upper limit rules do not guarantee the prevention of overfitting. Conversely, if the number of components were to be estimated based on the conventionally used 90–95% threshold of explained variance, the model order would have to be set to several 100 (see Figure 2B). Such a large estimate of model order will most likely lead to overfitting problems. Recent research (Abou-Elseoud et al., 2010; Allen et al., 2011) indicates that a model order around 70 components may represent an good heuristic estimate of model order to detect between-group differences and to avoid false positive results.

In order to avoid relying on prior model order assumptions given the divergent findings on model order estimation and the disparate model order selection approaches that currently exist, we aimed at decomposing our data into a varying number of assumed ICs, ranging from 10 to 100 in steps of 10, and examined the ensuing ICs derived from each decomposition.

### Independent component analysis (ICA)

ICA in its most general, noise-free form assumes that

(3) $$\begin{array}{l} X=AS, \end{array}$$

where the measured signal $X=\;{\left[x_{1},x_{2}\ldots x_{n}\right]}^{T}\in {\mathbb{R}}^{n}$ is a linear mixture of *N* statistically independent, non-normal, latent source signals $S=\;{\left[s_{1},s_{2}\ldots s_{n}\right]}^{T}\in {\mathbb{R}}^{n}$ which are indirectly observed and called the independent components (ICs), where *A*, referred to as the mixing matrix, is unknown. ICA attempts to find an unmixing matrix *W* ≈ *A*^−^ to recover all source signals, such that $WX=\hat{S}\approx S$, being the source signals optimized to be maximally independent. The rows of $\hat{S}\;$ are the recovered ICs, each of which represents temporally coherent functional networks, i.e., brain regions with synchronized source signals.

According to the above noise-free model (Equation 3), in our current dataset, *X* denotes the *N*-by-*V* group PCA-reduced matrix with *V* signals (voxels), and thus there are *N* instances of each signal. *A* is an *N*-by-*N* mixing matrix and *S* is a *N*-by-*V* matrix containing the *N* independent components. The rows of *S* are spatially independent images, and the columns of *A* are spatially independent time courses associated with those images. ICs were estimated via ICASSO, a robustness analysis tool that ensures stability of the estimated components (Himberg and Hyvärinen, 2003; Himberg et al., 2004). It accomplishes this by running the same ICA algorithm several times under different random initial conditions and bootstrapping, after which it performs clustering on the obtained estimations. ICA was run 100 times using the FastICA algorithm (Hyvärinen, 1999), known to yield consistent results for fMRI data analysis (Correa et al., 2007), with a maximum number of 100 different randomly initialized unmixing matrices up to convergence. The decorrelation approach used was symmetric, i.e., the estimation of all ICs was run in parallel, with hyperbolic tangent (*tanh*) as the set non-linearity. The rest of parameters were left as their defaults specified in FastICA. ICASSO was run for each model order. This yielded a set of group ICs consisting of a spatial map reflecting the ICs' functional connectivity pattern across space and their associate temporal courses reflecting the ICs' activity across time. The spatial maps were scaled by *z*-scoring and thresholded at *p* < 0.001 by means of a one-sample Wilcoxon signed rank test (*N* = 36, *p* < 0.001, cluster-wise corrected, FEW = 0.05) to test their mean values against the null hypothesis of no significant difference from zero. These group-level IC maps defined relevant networks at the group level for the whole participant pool.

Subject-specific IC temporal courses **(**${\tilde{R}}_{i}$) were then estimated via the back-reconstruction algorithm GICA3 (Erhardt et al., 2011), whereby the aggregate mixing matrix *A* ≈ *W*^−^ was back-projected to the subject space based on the PCA reducing matrices, such that

(4) $$\begin{array}{l} {\tilde{R}}_{i}=F_{i}{\left(G_{i}^{T}\right)}^{-}A, \end{array}$$

where ${\tilde{R}}_{i}\;$ is the *T*-by-*N* matrix of IC temporal courses corresponding to subject *i*, *F*_*i*_ is the *T*-by-*L* PCA reducing matrix corresponding to subject *i*, and $G_{i}^{T}$ is the *i* th subject partition of the transpose of *LM*-by-*N* group PCA reducing matrix *G*. GICA3 in combination with PCA reduction has been shown to produce accurate and robust results with the most intuitive interpretation in comparison to other back-projection procedures (Erhardt et al., 2011).

### Identification of pulse clarity-related components

To identify the components of interest, i.e., those associated with the stimulus' pulse clarity, a model of the pulse clarity of the musical stimuli implemented in MIR Toolbox (Lartillot and Toiviainen, 2007) was used. It is based on the autocorrelation of the amplitude envelope of the audio waveform, and conveys how easily the underlying pulsation in music can be perceived by the listeners (Lartillot et al., 2008). Lartillot et al. (2008) evaluated this model of pulse clarity by means of a perceptual test where participants rated the pulse clarity of musical excerpts. Thus, it is perceptually grounded, representing the clarity of the beat as perceived by listeners, where low pulse clarity denotes that the metrical pulsation cannot be perceived easily because it is not strong or clear enough. The relevant components were then selected based on the highest correlation coefficient between the ICs' associated time courses (derived from Equation 2) and the predicted waveform of the stimulus' pulse clarity. Spearman's rank correlation coefficient was chosen as the suitable non-parametric measure of statistical dependence since the potential relationship between pulse clarity and neural time courses may be monotonic, but not necessarily linear. The significance of the correlation coefficients had to be estimated due to the intrinsic serial correlation between adjacent fMRI samples, which reduces the effective degrees of freedom in the data. These were estimated by computing the cross-correlation between the participants' IC time course and pulse clarity (Pyper and Peterman, 1998). The estimate of effective degrees of freedom was averaged across participants per model order and subsequently used to compute the significance by dividing the Fisher *Z*-transformed correlation coefficients by the standard error $\frac{1}{\sqrt{df-3}}$, where *df* represents the effective degrees of freedom. *Z*-transformed correlation coefficients were corrected for multiple comparisons within each model order using the false discovery rate (FDR)-criterion (*q* = 0.05). The most significant component with a significance of at least *p* < 0.001 was retained per model order (Figure 6A shows the 10 most significant correlations per model order). Following this, Fisher's combined probability tests (Fisher, 1950; musicians and non-musicians combined, FDR corrected, *q* < 0.05) were performed to identify, for each model order, the best predicted IC by pulse clarity for the whole participant pool (see Figure 6B). The identification of one pulse clarity-driven IC per model order for the combined pool of musicians and non-musicians has the advantage of enabling statistical inferences to be drawn from group comparisons. Once the best predicted ICs were identified, two extra analysis were performed: (a) Fisher's combined probability tests for each group to determine whether significance was reached for both groups separately, and (b) non-parametric *t*-tests performed on the individual *Z*-scores (two-sample Wilcoxon signed rank tests) to test for significance differences between groups.

To assess the reliability of the pulse clarity-driven ICs, their ICASSO stability indices were retrieved. ICASSO stability (quality) index (*I*_*q*_; Himberg et al., 2004) is a criterion to validate the reliability and stability of ICA decomposition. It reflects the compactness and isolation of a cluster, which agglomerates similar ICs found in each ICASSO run. The *I*_*q*_ index scores the reliability of each extracted IC between zero and one. As the *I*_*q*_ approaches zero, it indicates that the IC is not reliable because its estimates from different ICA runs are not similar to each other. If it approaches one, the IC is reliably extracted, and therefore stable and robust.

### IC spatial maps associated with pulse clarity processing

The spatial maps associated with each IC temporal course were obtained by means of a one-sample Wilcoxon signed rank test (*p* < 0.001). To account for multiple comparisons, a non-parametric cluster-wise correction approach was used, whereby participants' IC spatial maps were bootstrap resampled with replacement from the pool of back-projected IC maps within a given model order (i.e., for model order *N*, 36 IC spatial maps were randomly drawn from a total of 36^*^N IC maps). The sample was then *t*-tested and thresholded (one-sample Wilcoxon signed rank test; *p* < 0.001). By running a sufficiently large number of iterations, a empirical distribution of cluster sizes was generated per model order. Bootstrap resampling within a given model order ensures not only that the spatial maps are uncorrelated, but also that the spatial autocorrelation structure is consistent among them. The maps were cluster-wise corrected using a FWE = 0.05.

### GLM analyses

For the purposes of comparing results between ICA and GLM, a voxelwise correlation analysis within the selected ROI was performed with pulse clarity separately for musicians and non-musicians to identify regions predicted significantly by it (Spearman's rho, *p* < 0.001, cluster-wise corrected, FWE = 0.05). We followed the same procedure of estimating the effective degrees of freedom explained in the previous section to correct the significance of the correlation coefficients, followed by a Fisher's combined probability test for each group (*p* < 0.001, cluster-wise corrected, FWE = 0.05).

## Results

### IC spatial maps associated with pulse clarity processing

**Figure 4:**
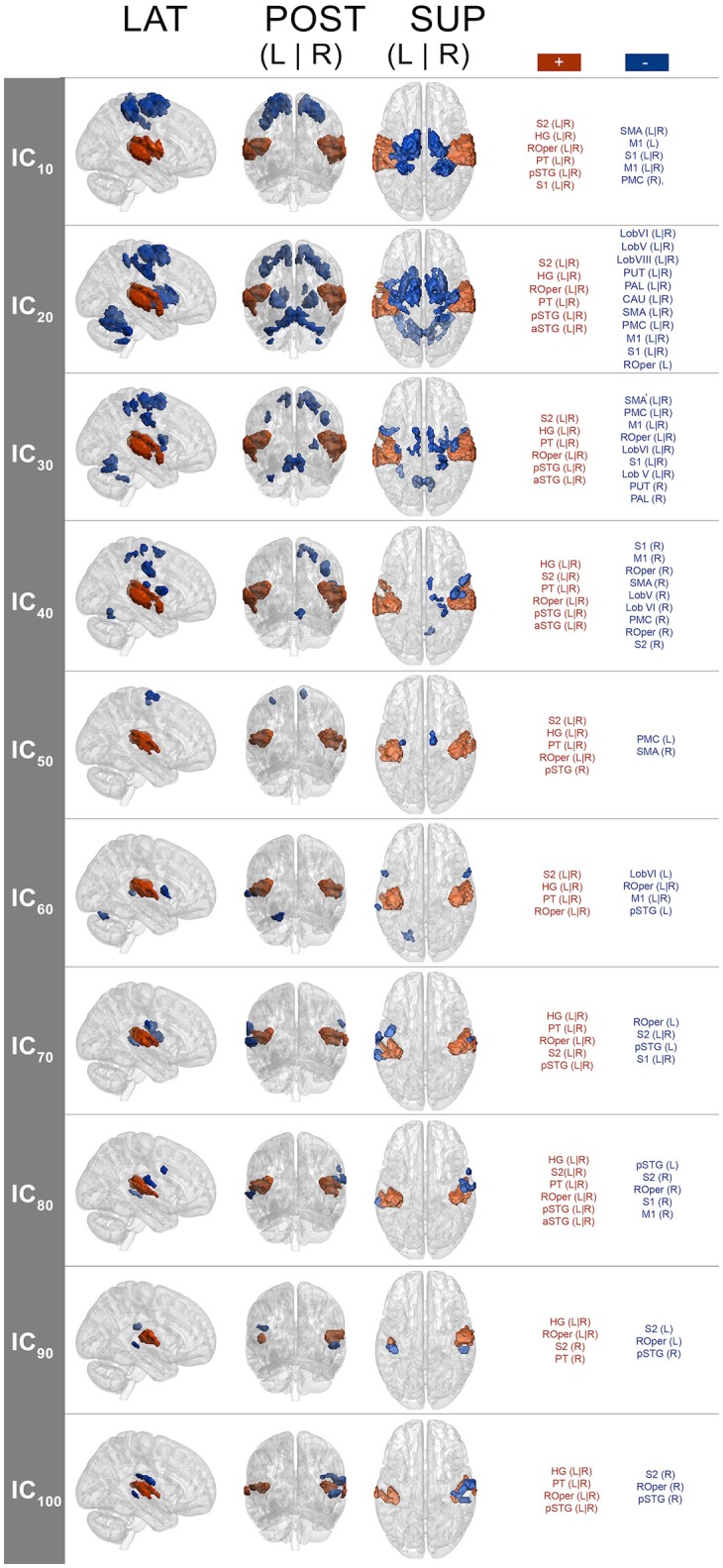
**Group-level IC spatial maps corresponding to IC temporal courses with maximal correlation with pulse clarity per model order (one-sample Wilcoxon signed rank test, *N* = 36, *p* < 0.001, cluster-wise corrected, FWE = 0.05)**. + (plus), positive correlation; – (minus), negative correlation; LAT, lateral view; POS, posterior view; SUP, superior view; L, left; R, right; S1, primary somatosensory cortex; S2, secondary somatosensory cortex; HG, Heschl's gyrus; PT, planum temporale; ROper, Rolandic operculum; pSTG, superior temporal gyrus (posterior); aSTG, superior temporal gyrus (anterior); PMC, premotor cortex.

The spatial maps (one-sample Wilcoxon signed rank test, *p* < 0.001, cluster-wise corrected, FWE = 0.05) corresponding to the pulse clarity-driven ICs are shown in Figure 4. Their ICASSO *I*_*q*_ indices showed an *I*_*q*_ > 0.90, except for IC_90_, with an *I*_*q*_ = 0.72, thus indicating that the ICs were reliably extracted. Overall, pulse-clarity networks were highly consistent across model orders in terms of polarity and topography, with auditory areas [Heschl's gyrus (HG), planum temporale (PT), and anterior and posterior superior temporal gyrus (aSTG and pSTG, respectively)] and somatomotor (M1, S1, S2, SMA, PMC, ROper) and CER areas exhibiting an inverse relationship. Generally auditory areas were positively associated with pulse clarity, whereas somatomotor areas and CER showed a negative association. Different sections of the ROper showed however both positive and negative relationships within the same IC spatial maps. The areas that were present in all ICs were the auditory cortices, ROper and S2, whereas large somatomotor areas were observed only in lower model orders. BG and CER were largely recruited only in low model order IC_20_.

### GLM analyses

**Figure 5:**
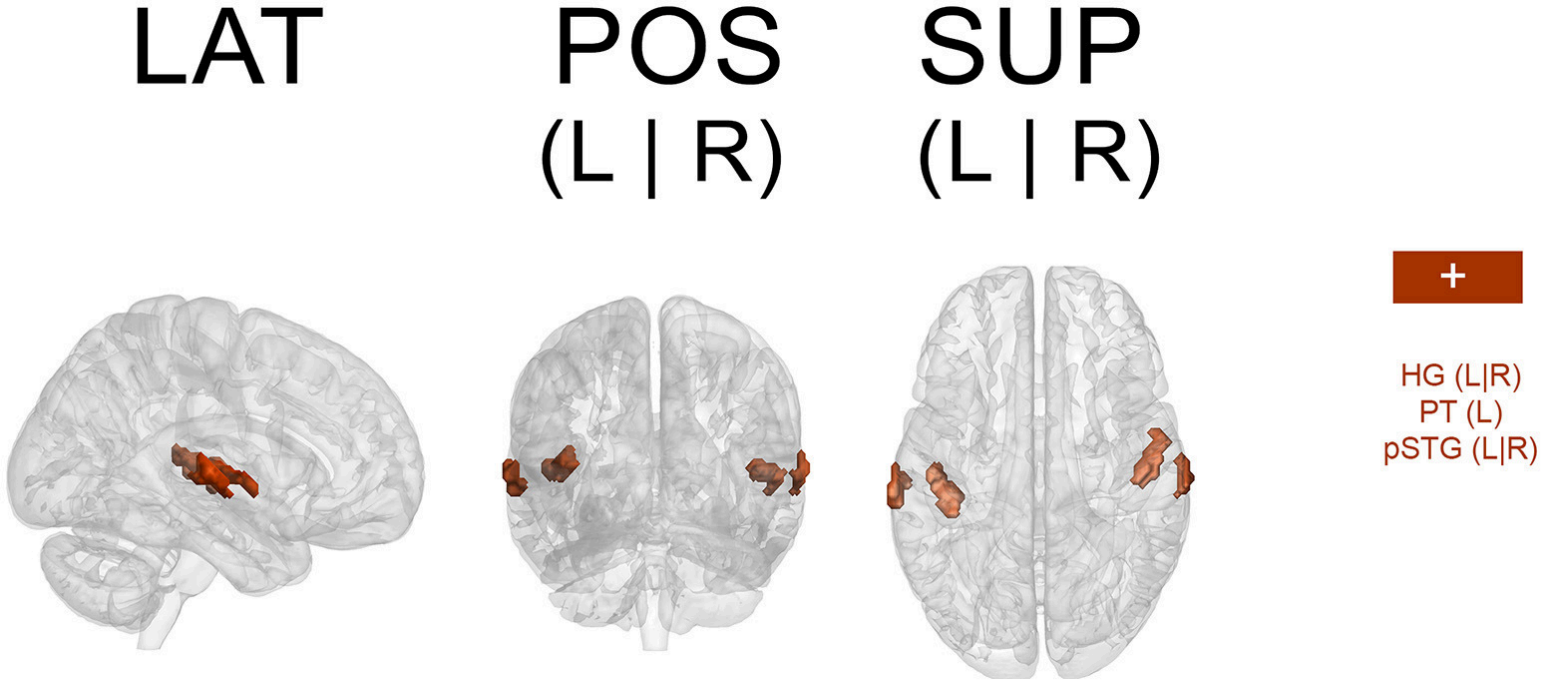
**Spatial map resulting from the GLM analysis (only non-musicians)**. Regions predicted by pulse clarity (Spearman's rho, *p* < 0.001, cluster wise corrected (FWE = 0.05). + (plus), positive correlation; – (minus), negative correlation; LAT, lateral view; POS, posterior view; SUP, superior view; L, left; R, right; HG, Heschl's gyrus; PT, planum temporale; pSTG, superior temporal gyrus (posterior).

Results from the GLM analyses (Spearman's rho, *p* < 0.001, cluster-wise corrected, FWE = 0.05) yielded significant results only for non-musicians and only for auditory areas (HG, PT, pSTG; see Figure 5). Results overlapped with those from the ICA analyses.

### Pulse clarity processing in musicians and non-musicians

**Figure 6:**
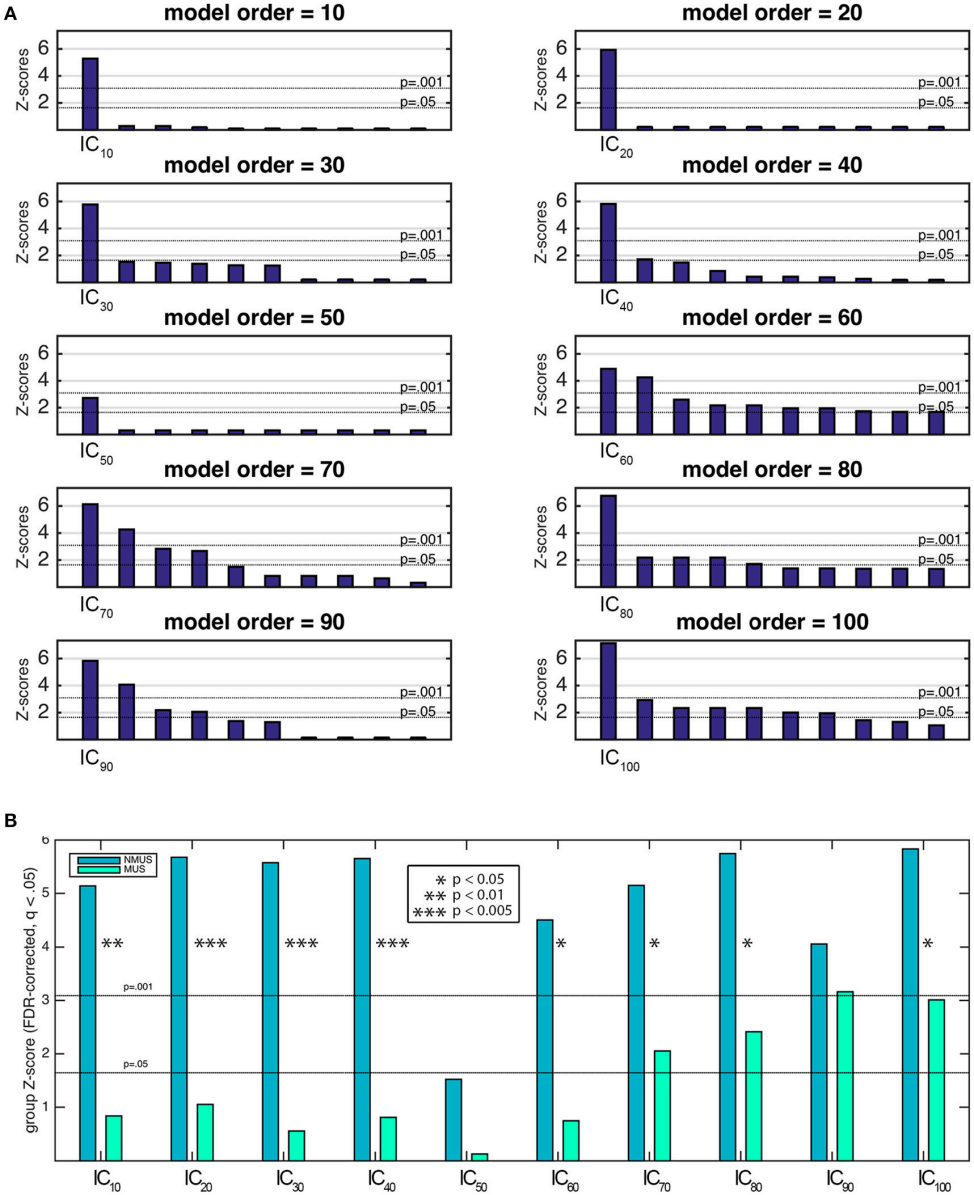
**(A)** Ten most significant correlations per model order resulting from the Fisher's combined probability test for the whole participant pool (musicians and non-musicians combined; Spearman's rho, FDR corrected, *q* = 0.05, within model order). **(B)** Musicians' and non-musicians' results from the Fisher's combined probability test (Spearman's rho, FDR corrected, *q* = 0.05, within model order). Additionally, significant between-group differences are indicated as a result of a nonparametric *t*-test performed on the individual *Z*-scores (Wilcoxon signed rank test).

Figure 6A shows the first 10 most significant correlations per model order between the temporal courses from each extracted spatial IC and pulse clarity for the whole participant pool (Fisher's combined probability test, FDR corrected, *q* < 0.05). For the purposes of group comparisons, we focused only the most significant IC driven by pulse clarity within model order that yielded a significance of at least *p* < 0.01 for the whole participant pool (in the following, IC_10_, IC_20_,…, IC_100_). Overall, for all ICs non-musicians' brain responses were notably better predicted by the pulse clarity of the music than musicians'. Non-musicians showed highly significant correlations at the group level for all pulse clarity-driven ICs (*p* < 0.0001) except for IC_50_ (*p* = 0.06), whereas musicians exhibited significant correlations only for the four highest model orders [IC_70_ (*p* < 0.05), IC_80_ (*p* < 0.01), IC_90_ (*p* < 0.001), and IC_100_ (*p* < 0.005); see Figure 6B]. Finally, the between-group comparisons (two-sample Wilcoxon signed rank tests) revealed significantly higher correlations for non-musicians compared to musicians for IC_10_ (*p* < 0.01), IC_20_ (*p* < 0.005), IC_30_ (*p* < 0.005), IC_40_ (*p* < 0.005), IC_60_ (*p* < 0.05), IC_70_ (*p* < 0.05), IC_80_ (*p* < 0.05), IC_100_ (*p* = 0.05), with IC_90_, showing a higher non-significant trend also in favor of non-musicians.

## Discussion

The aim of this study was to investigate the processing of an aspect of rhythm, namely clarity of the pulse (extracted computationally), during naturalistic music listening, and potential differences in pulse clarity processing between professional musicians and non-musicians. To this end, ICA was used to decompose participants' brain responses into ICs in a targeted hypothesis-driven ROI related to rhythm processing. An advantage of using a ROI-based ICA approach is that it improves the separation and anatomical precision of the identified spatial ICs as it includes in the analysis informative signals with respect to potentially interesting sources. The ICA decomposition was performed under a range of model orders, i.e., assuming a different number of sources (ranging from 10 to 100 in steps of 10) in order to avoid relying on prior model order assumptions. A total of 10 ICs (one per model order) were selected based on the highest correlation coefficient between IC temporal courses and a continuous measure of the pulse clarity of the music, obtained from the stimulus using computational acoustic feature extraction. Additionally, the associated spatial networks across model orders were examined.

### IC spatial maps associated with pulse clarity processing

Because non-musicians' brain responses at all model orders were overall significantly better predicted by pulse clarity than musicians' (see Section Pulse Clarity Processing in Musicians and Non-musicians), the associated spatial maps here discussed reflect to a higher degree the brain networks underpinning pulse clarity processing in non-musicians than in musicians.

The spatial maps associated with pulse clarity processing during continuous, real-world music listening revealed consistent action-perception networks across model orders. This observed consistency supports the idea that they respond to the same phenomenon (i.e., pulse clarity processing). The observed networks at lower model orders comprised auditory-motor areas, while at higher model orders they recruited mainly auditory areas. One feature of the observed networks was their consistent polarity for all decompositions. Positive sign was largely found for the auditory areas, whereas negative sign was observed for somatomotor and CER areas. This polarity may be construed as an action-perception functional network during pulse clarity processing, denoting an excitatory-inhibitory relationship. A tentative interpretation of this polarity may be that when the pulse is stable and clear in the music, auditory cortices engage as motor areas disengage, and when the pulse is less clear, the major engagement of the motor system, as auditory areas disengage, could respond to the demand to organize temporally complex auditory information. Although the question of this polarity remains unresolved, it is a relevant question and remains open for further study.

Interestingly, an aspect of this polarity, namely the activation of motor areas with decreasing pulse clarity, seems to be in disagreement with previous neuroimaging results on rhythm perception, which found increased regional activity in motor areas as rhythmic saliency increased (i.e., high pulse clarity). For instance, Bengtsson et al. (2009) found cortico-motor areas to be activated when listening to metrically less complex rhythm (isochronous sequence) compared to more complex sequences (non-metric and random) during a listening task. Similarly, Grahn and Brett (2007) observed that a simple rhythmic sequence elicited increased activity in BG and SMA to a greater extent than complex or non-metric rhythmic sequences. Moreover, in their study complex metric and non-metric sequences did not statistically differ in terms of their activation in all areas, which could mean that the contrast between these two conditions was not sufficient to observe significant differences.

We argue that previous studies assume motor-related activity to be a direct linear, or at least monotonic, function of complexity. However, the extent to which pulse prediction is engaged may exhibit an inverted *U*-shape as a function of rhythmic complexity, and such a continuum may not be captured by the stimuli used in controlled experiments. This is in line with previous work on groove (Witek et al., 2014) on movement propensity vs. rhythmic complexity (Burger et al., 2013), and more generally with aesthetic experience as a function of complexity (Berlyne, 1971, 1974; Nasar, 2002; Akalin et al., 2009). Thus, previous work may lack conditions that account for different degrees of rhythmic complexity (from simple to random sequences) that allow for increasingly challenging sequences. For instance, random sequences designed to represent highly complex rhythms are unpredictable. This may explain less involvement of BG or corticomotor activation than in simpler rhythms in previous work, as no predictions are available. A condition that represents a compromise between high predictability and unpredictability could show perhaps an increased activation of the motor system in response to increasing compared to decreasing complexity.

In addition, non-temporal aspects of the musical structure (i.e., melody, harmony, timbre, pitch) can impact the perception of pulse (Temperley, 1963; Dawe et al., 1993; Parncutt, 1994; Huron and Royal, 1996). This becomes relevant in real music, as it presents higher variability in a higher number of dimensions (i.e., dynamics, timbre, harmony, melody) than simple, controlled auditory stimuli. This characteristic of real music may facilitate the prediction of subsequent beats, even in highly complex sequences. Because this multidimensionality is missing in controlled stimuli, pulse tracking in controlled auditory conditions in may pose an additional challenge. Thus, the use of real music and a continuous measure of pulse clarity in our study may be one of the reasons for this discrepancy of results.

Furthermore, in contrast to previous work on rhythm processing based on mass-univariate analyses (GLM) targeted at findings regionally specific effects, ICA is a multivariate approach, which explicitly accounts for inter-regional dependencies. This makes multivariate inference more powerful than mass-univariate topological inference, because it does not depend on focal responses that survive a given threshold (Friston et al., 2008). Additionally, ICA seems to have a higher sensitivity for detecting task-related changes in fMRI signal compared to the widely used mass-univariate GLM-based approach as a consequence of a stricter criterion for spatial independence between spatial maps (ICs), which reduces noise in the final solution by separating artifactual and other physiological fluctuations from the fMRI signal of interest (McKeown et al., 1998). Similarly, because GLM-based approaches cannot segregate the signal mixture from each voxel into source signals, they are not suited to detect overlaps of functional networks and their temporal course modulation by cognitive tasks. ICA methods, conversely, are capable of disentangling signal mixtures. In this regard, how functional networks overlap with different temporal courses and their modulation by cognitive tasks is critical for understanding brain functional organization (Quintana and Fuster, 1999; Fuster, 2009).

If we consider the aforementioned observations, the discrepancy of our findings in view of previous work may be reconcilable as it may result from a combination of methodological factors. However, further research is required to determine the reason for this inconsistency of results.

Disregarding the polarity issue, previous research on rhythm processing found a similar network including BS areas, PMC, SMA, and auditory cortices responding to salient rhythms, which was observed in the current study at the model orders of 20 and 30 (see Figure 4) among other areas (such as S1, S2, ROper, M1, and CER). Conversely, at higher model orders, networks were mainly constrained to the auditory areas with minor encroachments into the S2 and ROper. This can be explained by the fact that at low model orders, signal sources tend to merge into singular ICs, which then split into several subcomponents at higher model orders (Abou-Elseoud et al., 2010). Thus, different choices of model order lead to the identification of different networks or subdivisions of networks (Kalcher et al., 2012).

There is a lack of knowledge on the neurophysiological reasons as to why some components tend to branch into more fine-tuned components while others remain stable. It is speculated that low model orders may group larger networks which are sparsely connected (van den Heuvel et al., 2008), whereas higher order would seem to group non-branching components which are more functionally independent from each other. Thus, low model orders may provide a general picture of large-scale brain networks (Abou-Elseoud et al., 2010). This hierarchical structure of functional brain networks would be organized in a highly efficient small-world manner (Sporns and Zwi, 2004; Stam, 2004; Achard et al., 2006), i.e., with a dense neighborhood clustering sensible to local information processing and sparse, long-distance connections in order to both target and integrate global communication across the network. At very high model orders, however, ICs' repeatability is known to decline (Abou-Elseoud et al., 2010). In the current study, the high quality index (*I*_*q*_) of all the pulse clarity-driven ICs guaranteed their stability and robustness, suggesting relatively good repeatability for all model orders. Moreover, between-group differences in functional connectivity measured with ICA might be affected by model order selection (Abou Elseoud et al., 2011). This was apparent in the current analyses, as group differences were more striking in low model orders (IC_10–40_) than in higher model orders (IC_60–100_; see Figure 6B). A hypothetical explanation of these results would be that, at low model orders, large-scale networks emerge which represent the functional footprint for pulse clarity processing specific of a particular population (e.g., musicians or non-musicians). Conversely, high model orders may uncover small-scale networks, which would constitute subcomponents or main functional hubs of the broader low model order networks. Accordingly, these main hubs may be conceived as more universal and hence characteristic of a wider population, common to individuals with and without professional musical training. In the current study, the auditory cortex would act as the main hub of the networks subserving pulse clarity processing, common to both musicians and non-musicians.

Given the trade-off between number of ROIs and resolution of the ICA solution, the current work was focused only on how action-perception networks sustain pulse clarity. Future work will use current findings to include additional regions, e.g., cortical areas associated with exogenous temporal expectation, so as to investigate top-down aspects of rhythm processing.

Finally, a key strength of the present approach was the inclusion of results from a continuum of model orders, rather than assuming a fixed number of sources, whereby different hierarchies are exposed in the functional brain organization of pulse clarity processing during continuous, real-world music.

### Comparison with GLM

The complementary GLM analyses were only significant for non-musicians, indicating that only non-musicians' brain responses to pulse clarity fitted the pulse clarity model used in the analyses. Thus, similarly to the ICA results, GLM results could be explained by the idea that musicians possess different models for predicting the pulse of the music. Furthermore, functional brain correlates underlying pulse clarity processing from the GLM approach evidenced a positive relationship between the stimulus' pulse clarity and non-musicians' auditory cortical activity, a result consistent with ICA analyses, especially at the higher model orders (see previous Section IC Spatial Maps Associated with Pulse Clarity Processing. IC spatial maps associated with pulse clarity processing). In sum, extra GLM analyses provided a framework against which to compare and validate the reliability of the ICA findings, and thus convergence of results denoted the robustness of the ICA approach. The complementary GLM analysis served as an additional reliability check, by demonstrating the power of the ICA approach, which enabled the detection of networks undetectable through GLM.

### Pulse clarity processing in musicians and non-musicians

Examination of the IC time courses across model orders indicated that non-musicians' brain activity was overall significantly better predicted by the stimulus' pulse clarity than musicians' (see Figure 6). Thus, the computational model of pulse clarity based on acoustical descriptors alone was insufficient in predicting the temporal evolution of activations to pulse clarity in musicians compared to non-musicians. These results would be in line with the notion that non-musicians' internal model of pulse clarity relies on the acoustical content of the stimulus to a greater extent than musicians', whose pulse clarity model would rely more on cognitive processes and top-down rules of metricality, facilitating enhanced internal beat generation. Tapping experiments indicate an advantage in synchronization abilities for musically trained individuals as opposed to controls (Drake et al., 2000; Hove et al., 2007; Repp and Doggett, 2007; Krause et al., 2010; Repp, 2010). These experiments indicate that musicians show smaller mean negative asynchrony (MNA; the tendency for taps to precede the pacing tones) than untrained individuals (Aschersleben, 2002). Supporting this, previous evidence highlights intense, lifelong musical training as an important factor influencing beat processing, either by enabling better predictions due to a stronger internal representation of the beat, via enhanced working memory abilities (Zatorre et al., 2010; Kung et al., 2011), or by creating a richer internal model stemming from explicit knowledge of musical rules (Grahn and Rowe, 2013). In sum, significant between-group differences may be attributed to the musicians' improved accuracy to internally keep the temporal regularities in the music. As such, this is a *post-hoc* explanation of the present results which would need further support from future experiments to determine its validity.

## Conclusion

The present study used a novel approach in the study of musical pulse processing by combining ROI-based ICA, a naturalistic auditory stimulation paradigm (free-listening to continuous real-world music), and acoustic feature extraction. The approach of relating brain responses during continuous music listening to computationally extracted acoustic features has been first applied by Alluri et al. (2012), replicated by Burunat et al. (2016) for fMRI, and by Poikonen et al. (2016a,b) for electroencephalography. Here, data decomposition at different assumed dimensionalities revealed the hierarchical organization of the functional networks subserving pulse clarity processing, hidden from GLM analyses. This networks exposed a strengthened functional action-perception network (auditory cortices, motor-related areas, BG and CER) consistent with previous neuroimaging work on rhythm processing. In addition, the fact that the associated spatial maps were spatially consistent across dimensionalities further supported the reliability of the approach. Results additionally revealed that non-musicians' internal model of pulse clarity relies on the acoustical content to a greater extent than musicians', which may be explained by musicians' improved predictive models of beat induction. These inferences are in line with evidence stressing intense musical training as a crucial factor that shapes beat processing.

## Author contributions

IB and PT conceived and developed the hypotheses, designed the analysis methodology of the study, and interpreted the results. VT contributed to the methodological design and performed the PCA reduction and preliminary ICA analyses. IB preprocessed the data, wrote the manuscript, performed ICA, subject back-reconstruction, and GLM analyses. EB coordinated the data collection, contributed to the design of the stimulation paradigm, the fundraising for the Tunteeet database, the preprocessing of the data, and obtained the ethics and research permissions. All authors revised the manuscript critically and approved the final version.

### Conflict of interest statement

EB is currently an associate Editor of Frontiers. The other authors declare that the research was conducted in the absence of any commercial or financial relationships that could be construed as a potential conflict of interest.
