# Supplementary material for: Coupling of Action-Perception Brain Networks during Musical Pulse Processing: Evidence from Region-of-Interest-Based Independent Component Analysis
Source: Front Hum Neurosci. 2017 May 9;11:230. doi: 10.3389/fnhum.2017.00230 (PMC5422442; doi:10.3389/fnhum.2017.00230)
Supplement: Supplementary file 1 [file DataSheet1.DOCX]

# **SUPPLEMENTARY MATERIAL**

## Details of the musical stimuli

Piazzolla Piazzolla, A. (1959). Adiós Nonino. [Recorded by Astor Piazzolla y su Sexteto]. On The Lausanne Concert [CD]. BMG Music. (1993)

Spotify link: <http://open.spotify.com/track/6X5SzbloyesrQQb3Ht4Ojx>

Excerpt: 0-08:07.968

Dream Theater Petrucci, J., Myung, J., Rudess, J. & Portnoy, M. (2003). Stream of Consciousness (instrumental). [Recorded by Dream Theater]. On Train of Thought [CD]. Elektra Records. (2003)

Spotify link: <http://open.spotify.com/track/3TG1GHK82boR3aUDEpZA5f>

Excerpt: 0-07:50.979

Stravinsky Stravinsky, I. (1947). The Rite of Spring (revised version for Orchestra) Part I: The Adoration of The Earth (Introduction, The Augurs of Spring: Dances of the Young Girls, Ritual of Abduction). [Recorded by Orchestra of the Kirov Opera, St. Petersburg - Valery Gergiev]. On Stravinsky: The Rite of Spring / Scriabin: The Poem of Ecstasy [CD]. Philips. (2001)

Spotify link: <http://open.spotify.com/album/22LYJ9orjaJOPi8xl4ZQSq> (first three tracks)

Excerpts: 00:05-07:52.243
